# Genetic variation, multiple paternity, and measures of reproductive success in the critically endangered hawksbill turtle (*Eretmochelys imbricata*)

**DOI:** 10.1002/ece3.1844

**Published:** 2015-11-23

**Authors:** Blanca Idalia González‐Garza, Adam Stow, Lorenzo Felipe Sánchez‐Teyer, Omar Zapata‐Pérez

**Affiliations:** ^1^ Centro de Investigación y Estudios Avanzados del Instituto Politécnico NacionalAntigua carretera a Progreso Km 6, Cordemex97310MéridaYucatánMéxico; ^2^ Department of Biological SciencesMacquarie University2109SydneyNew South WalesAustralia; ^3^ Centro de Investigación Científica de Yucatán A.C.Calle43 #130 Chuburna de Hidalgo97200MéridaYucatánMéxico

**Keywords:** Genetic diversity, Hawksbill turtle, mating system, multiple paternity, reproductive success

## Abstract

The Yucatán Peninsula in Mexico contains some of the largest breeding groups of the globally distributed and critically endangered hawksbill turtle (*Eretmochelys imbricata*). An improved understanding of the breeding system of this species and how its genetic variation is structured among nesting areas is required before the threats to its survival can be properly evaluated. Here, we genotype 1195 hatchlings and 41 nesting females at 12 microsatellite loci to assess levels of multiple paternity, genetic variation and whether individual levels of homozygosity are associated with reproductive success. Of the 50 clutches analyzed, only 6% have multiple paternity. The distribution of pairwise relatedness among nesting localities (rookeries) was not random with elevated within‐rookery relatedness, and declining relatedness with geographic distance indicating some natal philopatry. Although there was no strong evidence that particular rookeries had lost allelic variation via drift, younger turtles had significantly lower levels of genetic variation than older turtles, suggesting some loss of genetic variation. At present there is no indication that levels of genetic variation are associated with measures of reproductive success such as clutch size, hatching success, and frequency of infertile eggs.

## Introduction

Wide‐ranging, migratory species face multiple, often anthropogenic, challenges to their survival because they usually occupy different habitats to feed, rest, and breed (CMS 1983). The threat of environmental change is even more pronounced for those species with more specialized habitat requirements and smaller home ranges (Wilcove and Wikelski 2008). In addition, species with natal homing or spatially discrete breeding locations are more susceptible to the threat of inbreeding depression (Hudson 1998). Sea turtles are considered highly migratory and many species undergo transoceanic migration (Bowen et al. 1995; Bolten et al. 1998), occupy different habitats depending on their life stage (Spotila 2004), and typically show high site fidelity for egg laying and breeding location (Bowen and Karl 2007). Each of these characteristics render marine sea turtles especially vulnerable to anthropogenic impacts (Lutcavage et al. 1997).

The circumglobal distribution of most marine turtles means that environmental degradation and loss of habitat is likely to impact some part of their distribution (Pritchard 1997; Witherington et al. 2011). If these impacts reduce connectivity and population sizes, then, inevitably, genetic variation will be lost and this elevates the risk of extinction (Spielman et al. 2004) through loss of evolutionary potential and inbreeding depression (Frankham et al. 2002).

The rate at which genetic variation is lost in a particular species is a function of the effective population size, and this parameter is influenced by an organism's natural history and demographic history (Frankham et al. 2002). An evaluation of key parameters that shape effective population size is therefore needed to gauge the risk of genetic erosion and inbreeding depression (Allendorf et al. 2013). Fundamental to this is knowledge of both gene flow throughout a species' geographic distribution and the mating system. The mating system is relevant because the greater the reproductive skew, the lower the effective population size relative to the census size (Nunney 1993). Furthermore, the rate at which reduced effective sizes translate to inbreeding is mediated by mating behaviors such as inbreeding avoidance (Pusey and Wolf 1996; Stow and Sunnucks 2004; Skulkin et al. 2013). Inbreeding avoidance strategies could be precopulatory, including sex‐biased dispersal, mate choice (extra group copulation, avoidance of kin as mate), reproductive suppression and polyandry, or postcopulatory, such as sperm storage and competition, mate incompatibility and genetic incompatibility (Forster and Blouin 1988; Pusey and Wolf 1996; Ober et al. 1998; Tregenza and Wedell 2002; Dziminski et al. 2008).

For marine animals, reproduction is mostly mediated by spatial and temporal oceanographic processes, because organism‐specific ocean conditions are needed to promote migration, gonad maturation, spawning, fertilization, and embryonic development (Orton 1920; Pörtner and Peck 2010). Also, most marine species have high fecundity levels and high mortality at early stages, and therefore the number of offspring that will become the next generation of breeders is highly variable (Hedgecock 1994). This variation means that, on occasion, there is a reproductively successful minority, and the effective population size will be substantially smaller than the census size (Wright 1931; Newman and Pilson 1997).

In the case of sea turtles, when they are sexually mature, they commence moving from feeding grounds to specific breeding areas located close to their natal beach (Musick and Limpus 1997). Once females have mated, they move to their nesting beach to lay several clutches with an internesting period of approximately 15 days, during this period they remain nearby to avoid unnecessary energy expenditure (Zbinden et al. 2007a). Meanwhile, males stay at the breeding area to presumably copulate with more females and when the reproductive season ends, both, males and females, move back to their feeding grounds (Miller 1997). Direct observation of individual mating encounters is largely impractical and knowledge of mating systems has been reliant on the use of molecular markers.

**Figure 1:**
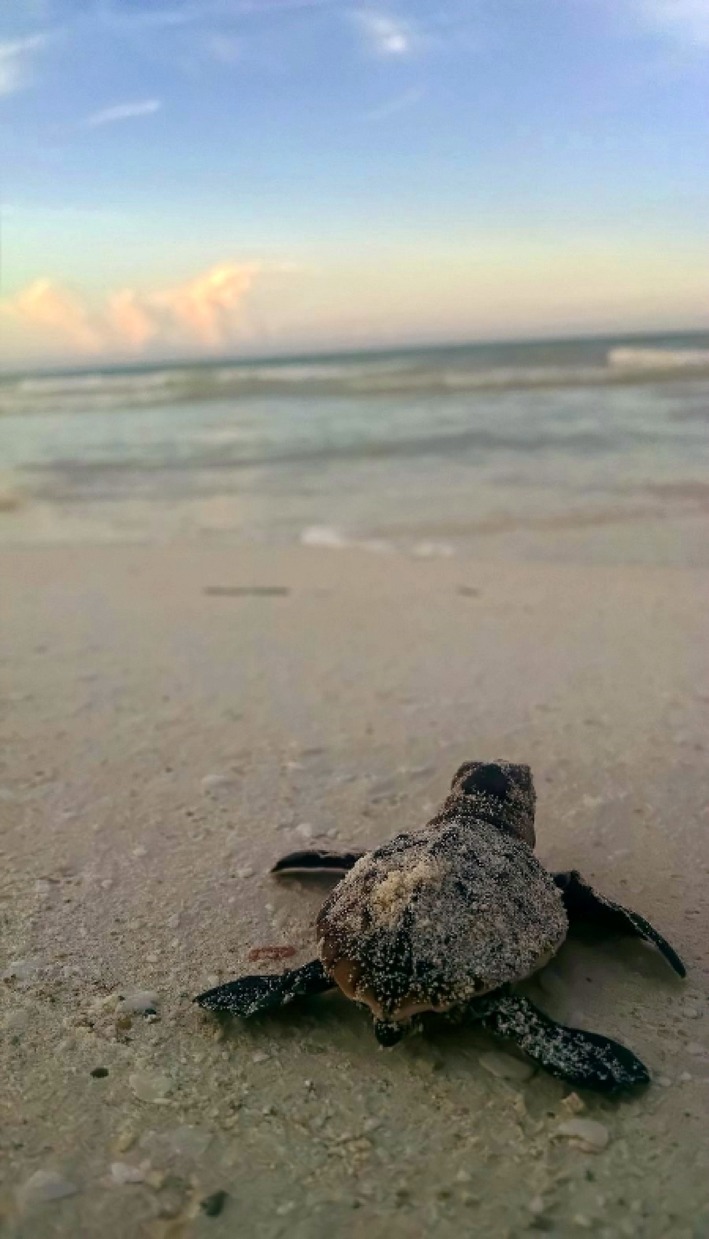
Hawksbill (*Eretmochelys imbricata*) hatchling crawling to the sea.

Molecular markers have been used in sea turtles to describe the mating system in the seven existing species, with multiple paternity and sperm storage characterizing six of them (*Chelonia mydas*,* Caretta caretta*,* Lepidochelys kempii*,* L. olivacea*,* Dermochelys coriacea,* and *Natator depressus*) (Pearse and Avise 2001; Bowen and Karl 2007; Lee 2008; Jensen et al. 2013). In contrast, the hawksbill turtle (*Eretmochelys imbricata*) (Fig. 1), which is categorized as Critically Endangered by the International Union for Conservation of Nature (Meylan and Donnelly 1999; Mortimer and Donnelly 2008), has been described in two studies as predominantly monogamous (Joseph and Shaw 2011; Phillips et al. 2013) with the ability to store sperm (Phillips et al. 2013). However, one of these studies was carried out at a single location in Malaysia (Joseph and Shaw 2011) and the other at a single location at the Republic of Seychelles (Phillips et al. 2013), and it has been suggested that multiple paternity levels in sea turtles could vary among locations and in accordance with population size (Bowen and Karl 2007; Lee 2008; Tedeschi et al. 2015).

The Yucatán Peninsula in Mexico harbors the largest hawksbill nesting population in the Atlantic and is considered among the most important in the world for the long‐term persistence of this species (Groombridge and Luxmoore 1989; Meylan 1999; Mortimer and Donnelly 2008). Satellite tracking of postnesting hawksbills from the Yucatan Peninsula showed that nesting females tend to remain in Mexican waters (González‐Garza et al. 2008; Cuevas et al. 2012). Although this could benefit the species by saving energy (e.g. low energy cost for short migrations distances), this also leads to concerns about inbreeding. Mitochondrial DNA analysis of hawksbill turtles in Mexican waters revealed that genetic variation is structured into two groups along the north and west coasts (Abreu‐Grobois et al. 2003). These data also indicate that juveniles on feeding grounds close to shore originated from beaches in the same region (Guzmán et al. 2008).

Here, we have microsatellite genotyped hawksbill hatchlings and their mothers from different nesting sites (rookeries) in the Yucatan Peninsula with the following objectives: (1) to assess the incidence of multiple paternity; (2) to test whether there are differences in genetic variation among rookeries; and (3) to evaluate whether individual levels of genetic variation are associated with measures of reproductive success.

## Methods

### Study locations and sampling

**Figure 2:**
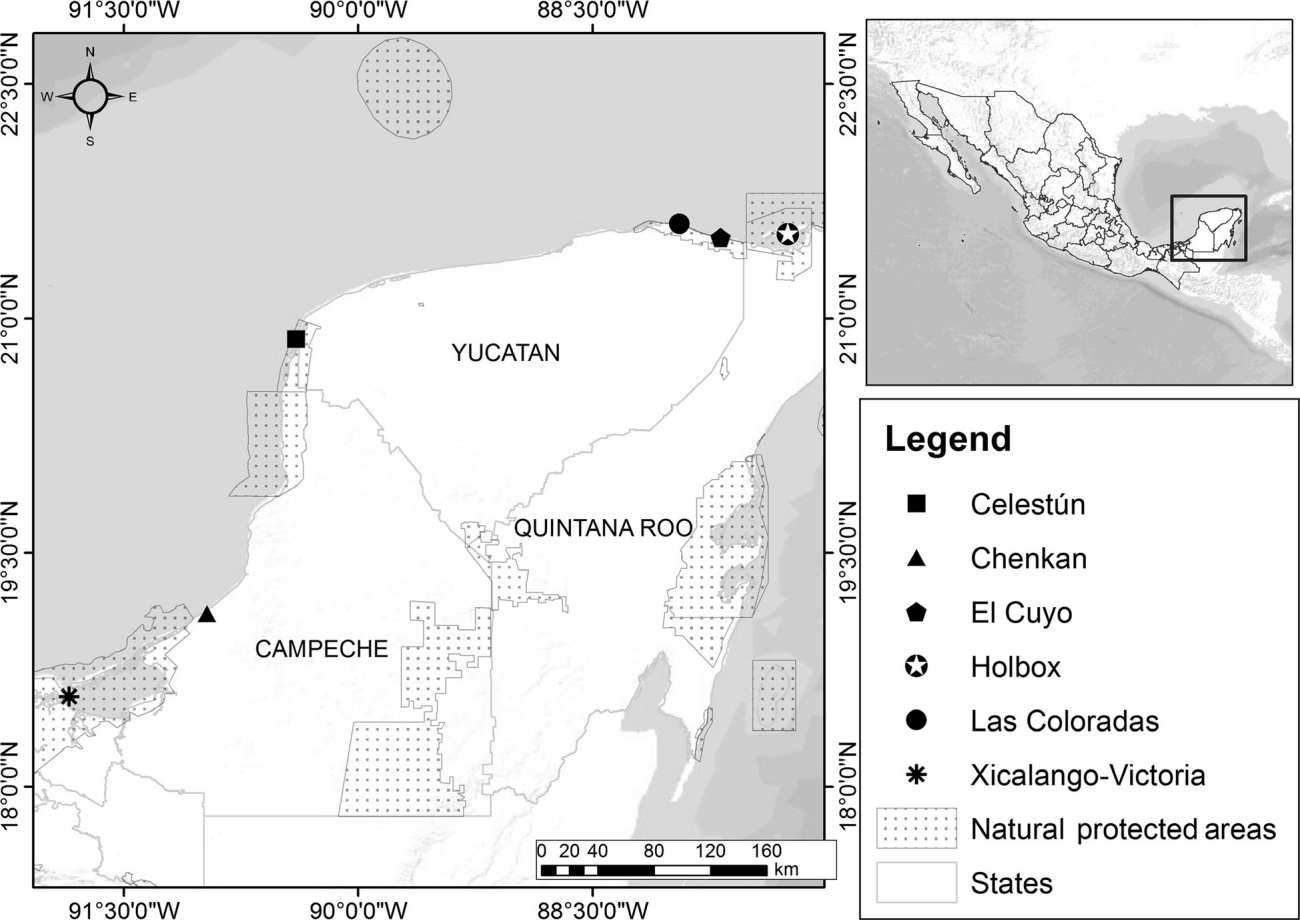
Location of the six hawksbill turtle rookeries on the Yucatan Peninsula, Mexico, which were sampled for this study.

We sampled six rookeries on the Yucatan Peninsula in Mexico: Xicalango‐Victoria and Chenkan in Campeche; Celestún, El Cuyo, and Las Coloradas in Yucatan; and Holbox in Quintana Roo, all of which are inside a Federal Natural Protected Area (Fig. 2).

During the 2011 nesting season, tissue samples were collected from the left rear flipper of 41 nesting females using a 3 mm biopsy punch. The tissue was preserved in 70% ethanol and stored at −4°C. Nest location coordinates were taken using a GPS and the nest location was marked with a stake placed nearby. After 55 days incubation, the nests were monitored daily and the hatchings were captured at emergence. Tissue samples were taken from the carapace left marginal scute using a 2 mm biopsy punch, and then preserved in 70% ethanol and stored at −4°C. After releasing the hatchlings, we were able to open the nest chamber of 44 clutches to record the number of shells and hatching live or dead hatchlings; unhatched eggs were also opened to determine the number of undeveloped and partially developed eggs.

### Molecular analysis

We extracted DNA using the DNeasy Blood and Tissue DNA extraction kit (Qiagen Inc., Valencia, CA) from a total of 1195 hatchling samples and 41 nesting female samples; for hatchlings, approximately 25 randomly selected samples were taken from each sampled clutch. DNA concentration was quantified using a Nanodrop 2000c spectrophotometer (Thermo Fisher Scientific Inc., Waltham, MA) and diluted to a standard concentration of 10 ng/*μ*L.

Thirteen previously designed polymorphic microsatellites for use in sea turtles were selected for genotyping: Ei8, Cm72, Cc117 (FitzSimmons et al. 1995); Cc141 (FitzSimmons et al. 1996); HKB22, HKB24, HKB25, HKB32 (Lin et al. 2008); Eim11, Eim31, Eim17, Eim12, Eim6 (Miro‐Herrans et al. 2008). The forward sequence of each microsatellite primer pair was fluorescently labeled using the DS‐33 dye set (Applied Biosystems, Foster City, CA); the dye used for each microsatellite sequence was selected depending on the expected size range of the fragment such that overlapping size range fragments had different colors.

Microsatellites were amplified using polymerase chain reaction (PCR) following the Invitrogen Taq DNA Polymerase, recombinant (Thermo Fisher Scientific Inc.) basic PCR protocol suggested by Innis et al. (1990). Thermal conditions for PCR comprised an initial denaturation for 5 min at 94°C, followed by 35 cycles of 94°C denaturation for 30 sec, 55°C annealing for 30 sec, 72°C extension for 1 min, and a 72°C final extension for 35 min; for primers Eim12, Eim31, and HKB24 annealing temperature was 58, 62, and 63°C respectively. The final extension time was increased to promote adenylation and to avoid ‐A peaks during genotyping. Products from PCR reactions were combined in three groups, and analyzed using an Applied Biosystem 3130 Genetic Analyzer and LIZ 600 as size standard (Applied Biosystems). Allele scoring was carried out using Genemapper v4.0 (Applied Biosystems), and allele calling was verified manually. Samples that failed to amplify at more than three loci were eliminated from the dataset.

### Multiple paternity

Observed and expected heterozygosity and the significance of any deviations from Hardy–Weinberg equilibrium were calculated from allele frequency data derived from the parent and one offspring from each of the analyzed clutches using GenAIEx v6.5 (Peakall and Smouse 2012). Loci were checked for the presence of null alleles using an iterative algorithm based on the observed and expected frequencies of the various genotypes (Dakin and Avise 2004), as well as the non‐exclusion probability for one candidate parent, in both cases using CERVUS (Marshall et al. 1998). The genotyping error rate was calculated for all loci by counting the number of unamplified samples and hatchlings with no maternal alleles and estimating the proportion of misleading data in the whole dataset.

Because we sampled the nesting females for 41clutches, and nine clutches were from already sampled females that laid successive clutches, we knew the maternal genotype with confidence and could consequently deduce the paternal alleles and reconstruct the paternal genotype (Jones 2001). In addition, we used the software COLONY v2.0 (Jones and Wang 2010), which uses a maximum‐likelihood method to reconstruct the genotypes of unsampled parents and assign parentage and sibship groups. COLONY's paternal reconstruction in clutches with multiple paternity was performed using a likelihood framework that calculates the probability of observing the genotypes of all offspring of an inferred father based on Mendelian expectations.

A total of 390 samples failed to amplify at more than three loci and were therefore excluded from this analysis. Offspring for which we had sampled the mother, but lacked the maternal allele(s), were also excluded and these data were used to estimate the genotyping error rate per locus. For the COLONY analysis the error rate ranged from 0.002 to 0.082. In COLONY, each rookery was analyzed as a single dataset using default parameters in a single medium‐length run and with medium full‐likelihood precision; the mating system for both sexes was considered polygamous, while an outbreeding model was selected because the data do not significantly deviate from Hardy–Weinberg equilibrium.

We calculated the probability of detecting multiple paternity for our sample size of 25 offspring per clutch using the software PrDM (Neff and Pitcher 2002). This software runs a model that assumes single‐sex multiple mating (i.e., polygyny or polyandry) and therefore all offspring in a brood are either full‐sibs or half‐sibs. We ran the software considering two sires per multiply mated brood, assuming several fertilization proportions ranging from 50–50% to 0.05–95%.

### Relatedness structure

We calculated relatedness using allele frequency data based on the sampled mothers, the reconstructed father genotypes, and one hatchling taken at random from each clutch. The software GenAIEx v6.5 (Peakall and Smouse 2012) was used to calculate the average pairwise relatedness of each rookeries using the Lynch and Ritland's (1999) estimator. In addition, we assessed the presence of spatial autocorrelation of genotypic similarity within several distance categories selected to provide equivalent sample sizes for each distance range analysed. The distance categories (in km) were as follows; 0–136; 137–272, 273–408, 409–544, 545–680. For both the relatedness analyses, and the assessment of spatial autocorrelation of genotypic similarity (*r*), the average relatedness for the whole data set is zero. We calculated the 95% confidence interval around zero using a permutation approach (999 permutations), and the 95% confidence interval around r were estimated by bootstrapping 999 times.

### Levels of genetic variation within each rookery

The observed and expected heterozygosity from each rookery and the significance of any deviation from Hardy–Weinberg Equilibrium was calculated in GenAIEx v6.5 (Peakall and Smouse 2012). To test for any signatures of inbreeding, measures of allelic richness (genetic variation accounting for differences in sample size) and *F*_IS_ were calculated using FSTAT (Goudet 1995), and individual multilocus homozygosity (HL) using STORM (Frasier 2008). The software STORM calculates observed HL, and then uses a Monte Carlo simulation to generate the expected distribution of these values according to the dataset (in this case, based on allele frequencies estimated for each rookery separately). The HL index weighs the contribution of each locus depending on their allelic variability and ranges from 0 to 1, being zero when all loci are heterozygous and 1 when all are homozygous (Aparicio et al. 2006).

### Genetic diversity and reproductive success

A Spearman correlation was carried out to evaluate whether there is a correlation between the HL index and estimates of reproductive success such as clutch size, hatching success, and number of fertilized eggs. To detect whether sea turtle age has an influence on genetic diversity indexes (HL), we used a Wilcoxon test to assess the index values between untagged nesting females, which are considered to be “young” (neophytes), first‐time nesters, and previously tagged nesting females, which are considered to be “older” (remigrant), experienced nesters.

## Results

### Descriptive statistics

We evaluated a total of 50 clutches, including some successive clutches, from 41 different females located at six different rookeries on the Yucatan Peninsula (Fig. 2). A total of 1195 hatchlings were successfully genotyped at 13 loci, with an average of 23.9 (±SD 2.3) hatchlings genotyped per clutch. Locus HKB22 was completely homozygous, and was therefore eliminated from the main dataset. The remaining 12 loci showed polymorphisms from three to 13 alleles per locus across all nesting groups, and a total of 87 alleles for the offspring dataset. The data pooled across rookeries did not significantly deviate from Hardy–Weinberg equilibrium (Table 1).

**Table 1:** Descriptive statistics of subsampled offspring and the 12 microsatellite markers. Number of analyzed clutches (*N*), number of different alleles (*N*_a_), observed heterozygosity (*H*_o_), expected heterozygosity (*H*_e_), Hardy**–**Weinberg equilibrium significance (HWE) (ns, not significant), inbreeding coefficient (*F*_IS_) and nonexclusion probability for one candidate parent given the genotype of a known parent of the opposite sex (NE‐2P), null alleles and error rate, and mean values (±SD) across all loci

Locus	*N*	*N*_a_	*H*_o_	*H*_e_	HWE	*F*_IS_	NE‐2P	Null alleles	Error rate
CC141	50	3	0.400	0.369	Ns	−0.073	0.842	−0.042	0.009
CM72	50	5	0.460	0.570	Ns	0.202	0.713	0.020	0.026
EIM12	50	10	0.800	0.845	Ns	0.064	0.307	−0.012	0.049
HKB24	50	7	0.560	0.564	Ns	0.016	0.669	−0.010	0.012
EIM11	50	10	0.780	0.825	Ns	0.065	0.341	−0.020	0.012
EIM31	50	10	0.760	0.728	Ns	−0.034	0.500	0.004	0.013
HKB25	50	3	0.080	0.078	Ns	−0.021	0.961	−0.025	0.009
CC117	50	9	0.720	0.743	Ns	0.041	0.473	0.007	0.047
EIM17	50	6	0.740	0.735	Ns	0.004	0.496	−0.24	0.012
EIM6	50	13	0.898	0.788	Ns	−0.129	0.392	−0.068	0.082
EI8	50	8	0.686	0.720	Ns	0.052	0.519	−0.031	0.026
HKB32	50	3	0.060	0.059	Ns	−0.014	0.971	−0.008	0.002
Across all loci		7.25 (±0.29)	0.57 (±0.28)	0.58 (±0.08)	Ns	0.01 (±0.08)	0.00093	0.004 (±0.04)	0.025 (±0.02)

### Multiple paternity

Using COLONY, we detected multiple paternity in 3 (6%) of the 50 clutches analyzed. These clutches with multiple paternity were located at the rookeries: Celestun, El Cuyo, and Holbox. There was no multiple paternity at Xicalango‐Victoria, Las Coloradas and Chenkan (Table 2).

**Table 2:** Parentage analysis and multiple paternity results obtained using COLONY

Rookery	Sampled clutches	Sampled females	Reconstructed males	Clutches with MP	% clutches with multiple paternity
Xicalango‐Victoria	2	2	2	0	0.00
Chenkan	16	10	10	0	0.00
Celestún	9	9	10	1	11.11
Las Coloradas	4	4	4	0	0.00
El Cuyo	12	10	11	1	8.33
Holbox	7	6	8	1	14.29
Total	50	41	45	3	6

**Figure 3:**
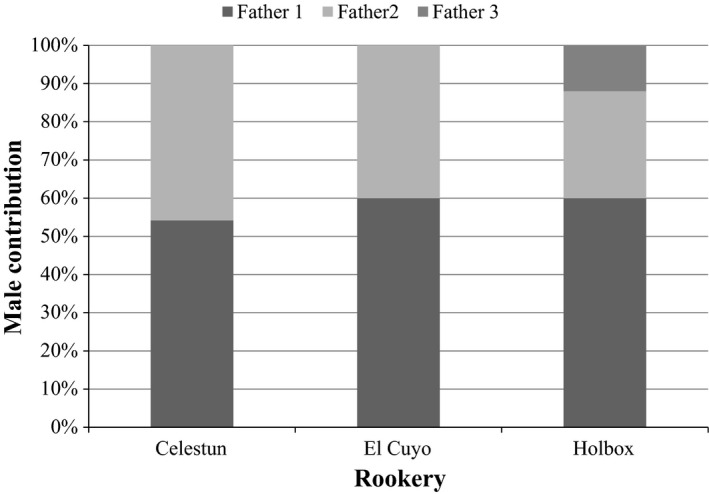
Relative contribution of different males to multiply sired clutches in three different hawksbill rookeries of the Yucatan Peninsula, inferred by COLONY.

The number of sires estimated for the clutches with multiple paternity was two for the clutches from Celestun and El Cuyo, while three sires were detected in the clutch from Holbox (Fig. 3). In all cases of multiple paternity, a single sire (the primary sire) tended to be responsible for the majority of fertilizations: 54–60%; while the secondary sire contributed 28–46% of fertilizations. For the clutch sired by three males, the third one contributed to 12% of the analyzed samples. The successive clutches laid by seven different females in three different locations did not show multiple paternity, and in all cases all clutches from a single female were fertilized by the same male.

Using the COLONY software, we accepted reconstructed paternal genotypes with the highest probability value (range of probability values = 0.80–0.99), and were ultimately able to reconstruct the genotypes of 45 different males that mated with 41 different nesting females. There were no matching genotypes among all the reconstructed male genotypes. For clutches with multiple paternity, each full‐sibling group was supported by private alleles from 4 to 6 loci. The probability of identity (probability of two independent samples having the same identical genotype), using all 12 microsatellites was very low (1.2E‐08 to 5.2E‐08). PrDM analysis estimates a high probability of 0.999 of detecting multiple paternity in our clutch sizes of 25 individuals, even when paternal contribution is skew to 90–10%, thus demonstrating that our results on multiple paternity are not likely to be strongly biased by incomplete sampling of clutches.

### Genetic variation within rookeries

**Figure 4:**
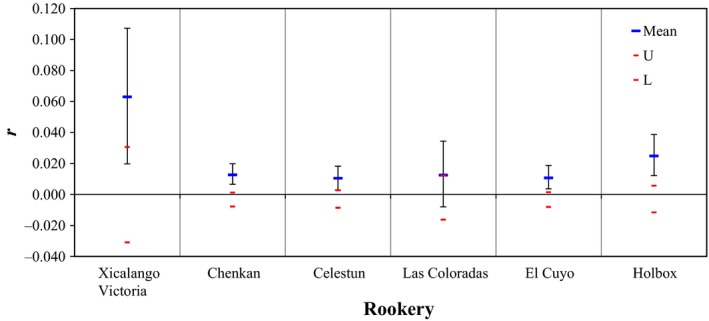
Mean Within Population Pairwise Values. Upper (U) and lower (L) confidence limits (red lines) bound the 95% confidence interval about the null hypothesis of no difference across the populations as determined by 999 permutations. The mean value that lies outside the 95% confidence interval indicates that relatedness for that population is elevated above the expected.

**Figure 5:**
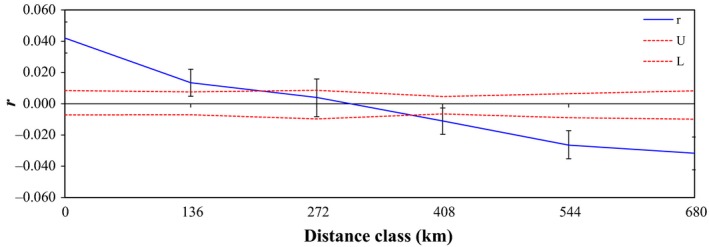
Correlogram plot of the genetic autocorrelation coefficient “r” as a function of geographic distance (Km). Upper (U) and lower (L) confidence limits (red lines) bound the 95% confidence interval about the null hypothesis of no spatial structure for the combined data set as determined by 999 permutations.

**Figure 6:**
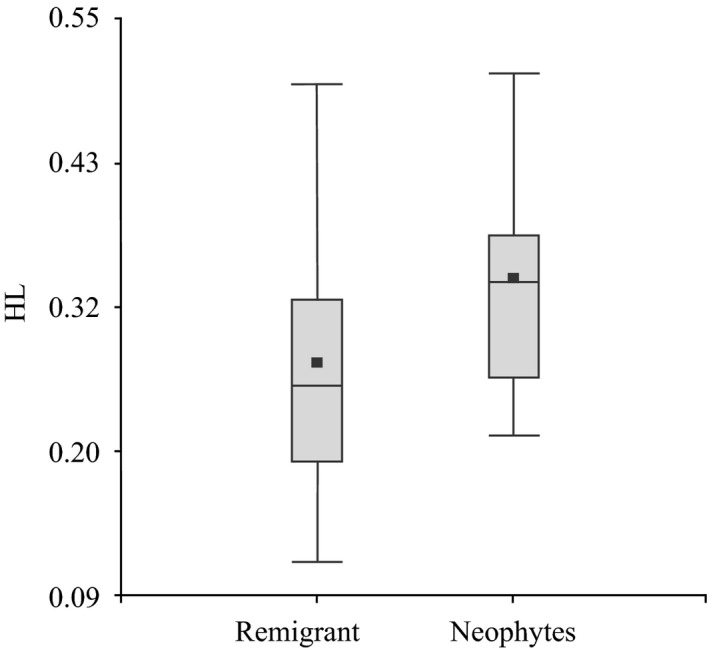
Homocigosity by loci (HL) values for offspring from “young” (neophites) (*N* = 21) and “old” (remigrant) (*N* = 28) nesting females.

Relatedness within each of the six rookeries was significantly greater than zero (Fig. 4). These results complement our finding of significant genotypic similarity within rookeries shown in the analysis of spatial autocorrelation. Spatial autocorrelation also showed that pairwise relatedness declines with increasing geographic distance (Fig. 5). Our analysis of Hardy–Weinberg equilibrium based on allele frequencies pooled across all rookeries show that, over time, dispersal is sufficient to prevent strong differences in allele frequencies (i.e. no significant deviation from Hardy–Weinberg Equilibrium, Table 1). However, it's clear that gene flow among rookeries is sufficiently limited to generate genotypic structure (relatedness structure). Consequently we analysed levels of genetic variation separately for each rookery to test for differences. We found that allelic richness does not vary significantly among rookeries (*H* = 0.77, *P* = 0.9788), *F*_IS_ does not significantly deviate from zero (Hardy–Weinberg Equilibrium). HL values vary among rookeries, but combined with the data on allelic richness and *F*_IS_, there is little evidence to suggest that higher HL is associated with loss of genetic variation (Table 3). However, there was significantly lower genetic variability (HL) in offspring from “younger” (neophytes) turtles than in those from the “older” (remigrant) ones (*W* = 597, *P* = 0.0252, Fig. 6). These data might point to a loss of genetic variation over time.

**Table 3:** Summary of genetic variation statistics per rookery. Sample size (*N*), Number of different alleles (*N*_a_), observed heterozygosity (*H*_o_), expected heterozygosity (*H*_e_), Hardy**–**Weinberg equilibrium significance (HWE) (ns = not significant), allelic richness (AR), inbreeding coefficient (*F*_IS_) and homozygosity by loci (HL)

Rookery	*N*	*N*_a_	*H*_o_	*H*_e_	HWE	AR^a^	*F*_IS_	HL
Xicalango‐Victoria	6	3.333	0.611	0.487	Ns	3.714	−0.167	0.50848
Chenkan	36	5.583	0.574	0.578	Ns	3.553	0.021	0.37333
Celestún	28	5.500	0.565	0.560	Ns	4.239	0.008	0.36482
Las Coloradas	12	5.250	0.660	0.594	Ns	3.586	−0.068	0.27675
El Cuyo	33	5.583	0.530	0.534	Ns	3.766	0.023	0.42011
Holbox	21	4.917	0.563	0.560	Ns	3.849	0.018	0.44608

a Ar based on a minimal sample size of 6 individuals.

### Genetic diversity and reproductive success

There was no evidence that genetic variation was related to differences in clutch size, hatching success, or percentage of infertile eggs, suggesting that levels of genetic diversity are not yet affecting any of these estimators of reproductive success.

## Discussion

Genetic variation was similar within each of the six rookeries examined on the Yucatán Peninsula, Mexico. While gene flow is sufficient to prevent significant differences in allelic variation between rookeries, genotypic similarity within rookeries is higher than random, and declines with geographic distance, suggesting some natal philopatry. Although individual rookeries did not vary in genetic variation, our finding that older females have higher genetic diversity than young ones, may indicate loss of genetic variation over time. Nonetheless, we found no indication that losses of genetic variation are at present detrimental, there was no relationship detected between our measures of genetic variation and reproductive success.

Microsatellite analysis showed that the incidence of multiple paternity in hawksbill turtles from the Yucatán Peninsula is low. The frequency of multiple paternity can vary between locations as a consequence of factors such as mate availability or inbreeding risk (Bowen and Karl 2007; Tedeschi et al. 2015). Our dataset reveals an overall frequency of multiple paternity (6%) that is lower to that previously reported for this species in the Seychelles (9.3%) and Malaysia (20%) (Joseph and Shaw 2011; Phillips et al. 2013). Given the wide geographic range of the various studies of multiple paternity in hawksbill sea turtles (i.e. the Atlantic, Indian and Pacific Oceans), we suggest that low multiple paternity rates are characteristic of the species, representing among the lowest multiple paternity rates recorded for any sea turtle (Table 4).

**Table 4:** Multiple paternity studies in all sea turtle species

Sea turtle species	Studies	Countries as study site	Total clutches analyzed	Total analyzed offspring (mean per clutch)	Overall % multiple paternity rate	Reference
Flatback (*Natator depressus*)	1	1	16	427 (26.7)	64	Theissinger et al. (2009)
Green turtle (*Chelonia mydas*)	9	6	230	5067 (30.9)	59	Peare and Parker (1996); Fitzsimmons (1998); Ireland et al. (2003), Lee and Hays (2004); Lara‐DeLa Cruz et al. (2010); Wright et al. (2012a,b); Ekanayake et al. (2013); Alfaro‐Núñez et al. (2015)
Kemp′s ridley (*Lepidochelys kempii*)	1	1	26	203 (7.8)	58	Kichler et al. (1999)
Olive ridley (*Lepidochelys olivacea*)	3	3	44	1457 (43.4)	54	Hoekert et al. (2002); Jensen et al. (2006); Duran et al. (2015)
Loggerhead (*Caretta caretta*)	7	4	216	5015 (24.7)	50	Harry and Briscoe (1988); Bollmer et al. (1999); Moore and Ball (2002); Zbinden et al. (2007b); Sakaoka et al. (2011); Lasala et al. (2013); Tedeschi et al. (2015)
Leatherback (*Dermochelys coriacea*)	4	2	79	2197 (18.9)	13	Rieder et al. (1998); Dutton et al. (2000); Crim et al. (2002); Stewart and Dutton (2011)
Hawksbill (*Eretmochelys imbricata*)	3	3	103	3145 (23.2)	11	Joseph and Shaw (2011); Phillips et al. (2013); Gonzalez‐Garza et al. present study.

Paternal contribution in the clutches with multiple paternity was mostly dominated by a single sire, which is typical of sea turtles (Birkhead and Hunter 1990), although in the two sired clutches paternal contribution were close to an equal contribution. It has been suggest that a low male contribution to a clutch could be the result of poor competitor sperm, or residual sperm stored from a previous nesting season (Stewart and Dutton 2011). In contrast, stored sperm within and across years has been reported as having the same hatchling success rate as clutches sired by newly acquired sperm (Pearse and Avise 2001; Uller and Olsson 2008). Therefore, it is not possible to determine whether variation on paternal contributions are the result of multiple mating on the same nesting season or sperm storage. Our data from successive clutches from the same female identified the same father siring all the successive clutches, this is consistent with other observations that females do not re‐mate during the internesting period, and that mating with one male is sufficient for successful fertilization of the eggs that a female will lay during an entire reproductive season (Fitzsimmons 1998; Pearse and Avise 2001; Stewart and Dutton 2011; Phillips et al. 2013).

High within‐rookery relatedness and declining pairwise relatedness with geographic distance indicates that there are different reproductive groups. It has been suggested that male hawksbills, like females, exhibit natal homing and site fidelity to breeding areas (FitzSimmons et al. 1997; Hamann et al. 2003; Bowen and Karl 2007; Lohmann et al. 2013). Therefore, they do not move far from the nesting areas, concentrating their breeding efforts on a specific area (van Dam et al. 2008; Phillips et al. 2013), and in turn, this might increase mating pair relatedness (Shields 1982). However, our data provide no strong evidence of regular inbreeding, *F*_IS_ values calculated with respect to allele frequencies for each rookery independently, and overall, do not deviate significantly from zero.

There may be some loss of genetic variation overtime, genetic diversity indices were higher for neophyte females, indicating that first‐time nesters produce offspring with lower genetic diversity. In the past, hawksbill turtles were abundant on the Yucatan Peninsula (Márquez 1978), but in 1968, more than 70% of sea turtle products worldwide originated from Mexico (Groombridge and Luxmoore 1989), with the hawksbill being among the most exploited species. The consequence of this overexploitation was a dramatic decrease in the number of nesting females in the region (Márquez 1996; Garduño‐Andrade et al. 1999). Such a decrease in the breeding sea turtle population should lead to an increase in genetic drift, and consequently a loss of genetic variation. Turtles are long‐lived (Gibbons 1987), and consequently lower genetic variation may only be reflected in younger turtles. An alternative explanation involves behavioral differences with respect to age, where older individuals show less natal homing, and as a consequence produce more genetically diverse offsprings by mating with males from other rookeries.

Despite some evidence for loss of genetic variation, we did not find any correlation between homozygosity by loci and clutch size, hatching success or percentage of infertile eggs, suggesting that current levels of genetic diversity are not affecting reproductive success on the Yucatán Peninsula. Despite evidence for some natal philopatry to rookery areas, gene flow among rookeries has been sufficient to prevent significant differences in allele frequencies. Thus, any loss of genetic variation through drift appears to be operating at a scale that at least collectively incorporates each of the rookeries we sampled.

## Conflict of Interest

None declared.

## Data Accessibility

Female genotypes, hatchling genotypes and reconstructed male genotypes: DRYAD entry doi: 10.5061/dryad.28v1b.

## Supporting information


**Appendix S1.** Sequence of previously designed microsatellite primers used in this work.
