# Supplementary material for: Genetic variation, multiple paternity, and measures of reproductive success in the critically endangered hawksbill turtle (Eretmochelys imbricata)
Source: Ecol Evol. 2015 Nov 23;5(24):5758–69. doi: 10.1002/ece3.1844 (PMC4717338; doi:10.1002/ece3.1844)
Supplement: Supplementary file 1 — Appendix S1. Sequence of previously designed microsatellite primers used in this work. [file ECE3-5-5758-s001.docx]

| Locus | Motif | Primer sequence 5´-3´ | Size Range (bp) | Ta °C | Author |
| --- | --- | --- | --- | --- | --- |
| Eim11 | (GATA)_13_ | F: GTTGGTGGAAGGAGTAAGTTT | 216-244 | 55 | Miro-Herrans et al. 2008 |
|  |  | R: CCCGAATCCCAATAGG |  |  |  |
| Eim31 | (GT)_17_ | F: ATCTGACTTGGGTGTGCATAC | 314-342 | 62 | Miro-Herrans et al. 2008 |
|  |  | R: ATCAGCTCCAGGTGTCCTAA |  |  |  |
| Eim17 | (GT)_17_ | F: TGGGAGGGTCAATGGT | 266-292 | 55 | Miro-Herrans et al. 2008 |
|  |  | R: CCTCCTTACAATGATACATGG |  |  |  |
| Eim12 | (GT)_13_ | F: GGCATCCTATAAGTGACCAC | 305-347 | 58 | Miro-Herrans et al. 2008 |
|  |  | R: GGATTACTCTCCTGTAGCCAT |  |  |  |
| EIm6 | (CT)_14_ | F: TCACTATTAAGGTCCCGACAT | 374-396 | 55 | Miro-Herrans et al. 2008 |
|  |  | R: CTCCCCCAATGCCTATATAA |  |  |  |
| HKB22 | (CA)_8-_(GA)_10_ | F: TACTCGAAACCTGGACAAAATGG | 202-260 | 55 | Lin et al. 2008 |
|  |  | R: AGAAGAAGTGGGCCTGGTGAG |  |  |  |
| HKB24 | (TG)_15_ | F: ATATTACCTCACCTGCCTTGTTTC | 72-172 | 63 | Lin et al. 2008 |
|  |  | R: TGTTCTACCAGTATGACGCACAC |  |  |  |
| HKB25 | (TA)_3_(CA)_8_(TA)_7_ | F: TTGCAGCCAACATGGAGGAAT | 350-366 | 55 | Lin et al. 2008 |
|  |  | R: ATTGGCTGCAGGTGTTCTAATCAG |  |  |  |
| HKB32 | (AC)_8_ | F: ACTCTAAATACACACAC | 94-120 | 55 | Lin et al. 2008 |
|  |  | R: CTTCATCAGTTGGCATGGTT |  |  |  |
| Ei8 | (CA)_19_ | F: ATATGATTAGGCAAGGCTCTCAAC | 170-250 | 55 | FitzSimmons et al. 1995 |
|  |  | R: AATCTTGAGATTGGCTTAGAAATC |  |  |  |
| Cm72 | (CA)_33_ | F: CTATAAGGAGAAAGCGTTAAGACA | 230-300 | 55 | FitzSimmons et al. 1995 |
|  |  | R:CCAAATTAGGATTACACAGCCAAC |  |  |  |
| Cc117 | (CA)_17_ | F: TCTTTAACGTATCTCCTGTAGCTC | 210-270 | 55 | FitzSimmons et al. 1995 |
|  |  | R: CAGTAGTGTCAGTTCATTGTTTCA |  |  |  |
| Cc141 |  | F: CAGCAGGCTGTCAGTTCTCCA | 180-210 | 55 | FitzSimmons et al. 1996 |
|  |  | R: TAGTACGTCTGGCCTGACTTTC |  |  |  |

**Appendix.** Sequence of previously designed microsatellite primers used in this work.
